# Lactation-related dynamics of bacterial and fungal microbiomes in feces of sows and gut colonization in suckling and newly weaned piglets

**DOI:** 10.1093/jas/skae321

**Published:** 2024-10-26

**Authors:** Fitra Yosi, Frederike Lerch, Julia C Vötterl, Simone Koger, Doris Verhovsek, Barbara U Metzler-Zebeli

**Affiliations:** Centre for Veterinary Systems Transformation and Sustainability, Clinical Department for Farm Animals and Food System Science, University of Veterinary Medicine Vienna, Vienna 1210, Austria; Christian-Doppler Laboratory for Innovative Gut Health Concepts of Livestock, Institute of Animal Nutrition and Functional Plant Compounds, Centre for Animal Nutrition and Welfare, University of Veterinary Medicine Vienna, Vienna 1210, Austria; Department of Animal Science, Faculty of Agriculture, University of Sriwijaya, Palembang 30662, Indonesia; Centre for Veterinary Systems Transformation and Sustainability, Clinical Department for Farm Animals and Food System Science, University of Veterinary Medicine Vienna, Vienna 1210, Austria; Christian-Doppler Laboratory for Innovative Gut Health Concepts of Livestock, Institute of Animal Nutrition and Functional Plant Compounds, Centre for Animal Nutrition and Welfare, University of Veterinary Medicine Vienna, Vienna 1210, Austria; Centre for Veterinary Systems Transformation and Sustainability, Clinical Department for Farm Animals and Food System Science, University of Veterinary Medicine Vienna, Vienna 1210, Austria; Christian-Doppler Laboratory for Innovative Gut Health Concepts of Livestock, Institute of Animal Nutrition and Functional Plant Compounds, Centre for Animal Nutrition and Welfare, University of Veterinary Medicine Vienna, Vienna 1210, Austria; Christian-Doppler Laboratory for Innovative Gut Health Concepts of Livestock, Institute of Animal Nutrition and Functional Plant Compounds, Centre for Animal Nutrition and Welfare, University of Veterinary Medicine Vienna, Vienna 1210, Austria; Centre for Animal Nutrition and Welfare, Clinical Department for Farm Animals and Food System Science, University of Veterinary Medicine Vienna, Vienna 1210, Austria; Clinical Centre for Population Medicine in Fish, Pig and Poultry, Clinical Department for Farm Animals and Food System Science, University of Veterinary Medicine Vienna, Vienna 1210, Austria; Centre for Veterinary Systems Transformation and Sustainability, Clinical Department for Farm Animals and Food System Science, University of Veterinary Medicine Vienna, Vienna 1210, Austria; Christian-Doppler Laboratory for Innovative Gut Health Concepts of Livestock, Institute of Animal Nutrition and Functional Plant Compounds, Centre for Animal Nutrition and Welfare, University of Veterinary Medicine Vienna, Vienna 1210, Austria

**Keywords:** bacteriome, lactation, mycobiome, piglet, sow, weaning

## Abstract

Changes in the gut microbial composition of the sow during lactation may influence the gut microbial colonization in their offspring, for which less information was available in the literature. This study aimed to assess: 1) the changes that occur in the bacterial and fungal communities in sow feces during the 28-d lactation period as well as in gastric and cecal digesta of piglets until one week after weaning, and 2) bacterial and fungal taxa in cecal digesta of the piglets postweaning that associate with fecal consistency. Aside from sow milk, piglets had access to creep feed from day of life (**DoL**) 3. Fecal samples from sows for microbial analysis were collected (*n* = 20) on days postpartum (**DPP**) 1, 6, 13, 20, and 27, as well as from weaned piglets for fecal scoring on DoL 30 and 34. Gastric and cecal digesta of piglets was collected on DoL3, 7, 14, 21, 28, 31, and 35 (*n* = 5/sex/DoL). Progressing lactation affected bacterial and fungal communities in sow feces, including 10.3- and 3.0-fold increases in the relative abundances of *Lactobacillus* from DPP1 to 6 and *Kazachstania* from DPP1 to 13, respectively (*P* < 0.001). Although time- and gut-site-related differences existed, bacterial and fungal taxa found in sow feces were also present in gastric and cecal digesta of piglets, which supports their role in gut colonization in neonatal piglets. In piglets, bacterial and fungal alpha-diversities showed certain fluctuations during the suckling period, whereby weaning affected the fungal than bacterial diversity at both gut sites (*P *< 0.05). At both gut sites, *Lactobacillus* largely increased from DoL3 to 7 and remained a dominating taxon until DoL35 (*P* < 0.05). Postweaning, plant-glycan fermenters (e.g., *Prevotella*-9) seemed to replace milk-glycan fermenting *Fusobacterium* and *Bacteroides* (*P* < 0.05). In gastric and cecal digesta, *Kazachstania*, *Tausonia*, *Candida,* and *Blumeria* were dominating fungi from DoL3 to 35, with *Kazachstania* becoming even more dominant postweaning (*P* < 0.001). Fecal consistency was softer on DoL34 than 30 (*P* < 0.05). Correlation analysis identified that softer feces were linked to the relative abundances of plant-glycan and proteolytic bacterial taxa including pathobionts (e.g., *Clostridium* sensu stricto) in the cecum on DoL34. However, the potential association between cecal mold and plant-pathogenic fungi *Talaromyces*, *Mrakia*, and *Blumeria* and softer feces are worth investigating in the future in relation to (gut) health of piglets.

## Teaser

We investigated the bacterial and fungal communities in the feces of sows during lactation which appeared to have contributed to the gut colonization of suckling piglets. Postweaning, we could identify taxa that were linked to firm and softer feces and may serve as bacterial and fungal markers for the gut homeostatic condition.

## Introduction

Postnatal microbial colonization is a substantial contributor to gut maturation, including immune and barrier functions (Everaert et al., 2017). Microbes that colonize the piglet’s gastrointestinal tract after birth originate from the sow (i.e., vagina, skin/nipple surface, colostrum/milk, and feces), environment, and handling farm staff (Jost et al., 2014; Chen et al., 2018). During the suckling period, piglets are commonly housed in farrowing pens with their sows until weaning (Nowland et al., 2019). Hence, changes in the gut microbial composition of the sow that occur during lactation may influence the gut microbial colonization in their offspring. Despite increasing knowledge on the gut microbial development in piglets, alterations in the gut microbial composition of the dam over the course of lactation have rarely been investigated so far.

Moreover, knowledge of the postnatal fungal colonization of the piglet’s gut and whether this development is driven by the fecal mycobiome of the sow is still scarce. The stomach of suckling and weaned piglets contains a diverse and complex microbiome (Mann et al., 2014; Lerch et al., 2023), which contributes to the degradation of nutrients and the production of fermentation metabolites. Although some data for the postnatal development of bacteria in the stomach exists (Lerch et al., 2023), the development of gastric fungal communities has been poorly described. Evidence from our group supports the importance of investigating fungal communities in the neonatal period (Lerch et al., 2023), as some of the fungi found in cecal digesta represented plant pathogens and mold fungi (Yeh et al., 2021), which may compromise the development of the immune system (Wang et al., 2023). This indicates the need for monitoring the development of the gastrointestinal microbial communities in healthy piglets from pre- and postweaning more closely.

Scoring the fecal consistency in piglets is also helpful for identifying eubiotic and dysbiotic gut conditions. Recently, we have shown that feces of different colors and consistencies differ in their bacterial composition in suckling and newly weaned piglets (Metzler-Zebeli et al., 2023). However, feces are mostly representative of the distal large intestine of piglets; therefore, targeting the microbial composition in the more proximal parts of the gut (e.g., cecum) and linking it to the fecal score may provide further marker taxa for eubiotic and dysbiotic gut conditions. In particular, the relationship between intestinal fungi and fecal score has been little investigated so far.

The objectives of this study were to assess 1) the changes that occur in the bacterial and fungal communities in sow feces during the lactation period as well as in gastric and cecal digesta of piglets from the day of life (**DoL**) 3 until one week after weaning; and 2) bacterial and fungal taxa in cecal digesta of piglets postweaning that associate with fecal consistency. Our research was based on the following hypothesis: 1) microbial communities in the piglet’s stomach and cecum would comprise similar bacterial and fungal taxa to that of the sow feces during the suckling phase but would diverge postweaning and 2) the association of fecal scores with microbial taxa in cecal digesta would allow the identification of taxa linked to a lower (dysbiosis) and higher (eubiotics) gut homeostatic state postweaning.

## Materials and Methods

### Ethical statement

All procedures requiring animal handling and treatment have been approved by the institutional ethics committee of the University of Veterinary Medicine Vienna and National authority in accordance with the Law for Animal Experiments in Austria (GZ 2020-0.437.208).

### Animals, housing, and experimental procedures

The pig experiment was conducted at the pig facility of the University of Veterinary Medicine Vienna (VetFarm) under production conditions, consisting of 2 consecutive replicate batches. In each replicate batch, 10 sows (Large White) and their litters (Large White × Piétrain) were used. The average litter size was 13.9 ± 1.7 (SD) alive piglets at birth across both replicate batches. The total number of piglets born alive was 143 and 134 in replicate batches 1 and 2, respectively.

The experiment lasted from 26 d prior to farrowing, throughout the 28-d lactation period to 7 d after weaning. At the pig facility, sows and their litters were handled according to standard procedures at the pig facility. Sows were group-housed in pens during gestation but had access to individual feeders. Five days before farrowing, sows were transferred to separate farrowing pens (BeFree, Schauer Agrotonic, Prambachkirchen, Austria; 2.3 × 2.6 m in size), which were equipped with a feeder, bowl drinker, and hayrack for the sow (for environmental enrichment and nesting behavior), as well as a round feeder, small bowl drinker, and nest with heated flooring for the piglets. All sows gave birth within 48 h and were not restrained during the farrowing process and the whole lactation period. Piglets were supplemented with iron by injection on DoL4 (2 mL of Ferriphor 100 mg/mL, OGRIS Pharma Vertriebs-GmbH, Wels, Austria), followed by castration of male piglets on DoL11 (general sedation with Stresnil 40 mg/mL, 0.025 mL/kg body weight, Elanco Tiergesundheit AG, Basel, Switzerland and Narketan 100 mg/mL, 0.1 mL/kg body weight, Vetoquinol Österreich GmbH, Vienna, Austria). On DoL17, piglets were vaccinated (1 mL Ingelvac CircoFLEX and 1 mL Ingelvac MycoFLEX, both from Boehringer Ingelheim GmbH, Ingelheim/Rhein, Germany). On DoL28, sows were removed from the farrowing pens and all remaining piglets were transferred to rearing pens in an outdoor climate house with a heated lying area. Each rearing pen had a size of 3.3 × 4.6 m each and was equipped with one round feeder, nipple and bowl drinkers, and a heated nest. The animal’s health was monitored daily throughout the experiment. Piglets with health problems were removed from the experiment. Water was freely available to sows and piglets throughout the experiment.

### Feed and feeding

The feeding protocol of gestating and lactating sows as well as of suckling and weaned piglets corresponded to the standard protocol at the pig facility. From 26 to 5 d prior to farrowing, sows were provided with a gestation diet in the morning (08:00 hours) and afternoon (14:30 hours; 3 to 4 kg/meal; Königshofer GmbH, Ebergassing, Austria; Supplementary Table S1 and S2). After moving the sows to the farrowing pens 5 d before the farrowing date, the sows were offered a lactation diet (approximately 3 kg/meal; Königshofer GmbH, Ebergassing, Austria; Supplementary Table S1 and S2) in the morning and afternoon (08:00 and 14:30 hours). Additionally, sows received 500 g of linseeds soaked in water once a day for 5 d prior to farrowing to avoid constipation. After farrowing, the feed amount of the sows was gradually increased (approximately 4 to 9 kg/meal) according to the regular feeding protocol.

Litters received creep feed that was manually prepared at least twice daily (08:00 and 15:00 hours) from DoL3 to 28. In the first 3 wk of life, the creep feed was a commercial milk replacer (NuriStart Sweet, BIOMIN Holding GmbH, Part of DSM-Firmenich, Getzersdorf, Austria; Supplementary Table S2), which was prepared according to the manufacturer’s instructions. The milk replacer was offered in liquid form, by mixing the powder with warm water (45 °C) at a ratio of 500 g/L (w/v) by hand. The milk replacer was always prepared freshly before feeding and the stainless steel feeders were cleaned before re-filling them. Each litter received a minimum amount of 1,000 mL per day (500 mL at 08:00 and 500 mL at 15:00 hours), and more when piglets finished their portion. From DoL3 to 23, the piglets were given 100% of milk replacer. Then, the milk replacer was gradually mixed with the prestarter diet from DoL24 to 26 (Königshofer GmbH, Ebergassing, Austria; Supplementary Table S1 and S2), starting with a ratio of 70:30 (w/w) on DoL24, 50:50 (w/w) on DoL25, and 30:70 (w/w) on DoL26, respectively, and provided in mash form. After that, the litters were fed 100% of the prestarter diet as mashed on DoL27 and in dry form after weaning from DoL28 to 35. All diets used in the study met or exceeded the current recommendations for nutrient requirements (NRC, 2012). Leftover creep feed and spills were collected to estimate the creep feed intake during the suckling phase. The feed intake after weaning could not be estimated due to the set-up of the round feeders in the rearing barn.

### Body weight measurement, fecal score, and collection

Piglets were weighed at 8 time points, immediately after birth, DoL4, 6, 13, 20, 27, 30, and 34. Freshly defecated feces of sows or feces after rectal stimulation were collected on days postpartum (**DPP**) 1, 6, 13, 20, and 27 for microbiome analysis. To avoid contamination from the floor, only the inside part of the defecated feces was used and homogenized with a sterile spatula before placing them into cryo tubes. The tubes were kept on ice before storage at −80 °C until further analysis. Fecal samples from weaned piglets were collected by means of rectal stimulation on DoL30 and DoL34 to assess fecal consistency. To obtain the samples, the inner anal sphincter was stimulated by inserting a sterile cotton tip and gently rotating it. The consistency was scored according to 0 (hard and dry balls), 1 (clearly defined shape with cracks), 2 (pasty; moist feces with no cracks), 3 (very wet but not liquid feces), 4 (liquid feces with minimal consistency), and 5 (entirely liquid feces). Fecal scores of 4 and 5 were considered as clinical signs of diarrhea.

### Gut sampling

In each of the 2 replicate batches, gut sampling took place on DoL3, 7, 14, 21, 28, 31, and 35. On each sampling day, 5 female and 5 male piglets were used for invasive sampling in each replicate batch. From each litter, 1 piglet (with alternating sexes on the consecutive sampling days) was selected per sampling day based on having average body weight within the litter. Prior to slaughter, piglets were weighed and anesthetized in the ear vein with azaperone (Stresnil 40 mg/mL, 0.025 mL/kg body weight, Elanco Tiergesundheit AG, Bad Homburg, Germany) and ketamine (Narketan 100 mg/mL, 0.1 mL/kg body weight, Vetoquinol Österreich GmbH, Vienna, Austria). Afterward, piglets were euthanized with embutramide (T61, 0.1 mL/kg body weight, Intervet GesmbH, Vienna, Austria) via intracardiac injection. Piglets were bled by cutting the neck. Then, the abdomen was opened and the entire gut was removed aseptically. The stomach and cecum were identified, clamped, and separated. Digesta samples were then collected from both gut segments. Homogenized digesta samples for microbiome analysis were snap-frozen in liquid nitrogen and stored at −80 °C.

### DNA extraction, 16S rRNA and ITS sequencing, and bioinformatics

For DNA extraction, the protocol as described in Lerch et al. (2023) was used (DNeasy PowerSoil Pro Kit; Qiagen, Hilden, Germany) with the same modifications including a heating step and mechanical lysis. The concentration of DNA in each extract was measured with a Qubit fluorometer (Qubit 4 Fluorometer, Thermo Fisher Scientific, USA) using the Qubit dsDNA HS Assay Kit (Thermo Fisher Scientific Inc., Waltham, MA, USA). Targeted 16S rRNA gene (V3-V4 hypervariable region) and ITS2 amplicon sequencing were performed in an external laboratory (Novogene, Cambridge, UK). In order to do so, aliquots of the DNA extracts were sent for library preparation (NEBNext Ultra II DNA Library Prep Kit, Illumina, San Diego, CA, USA). The 16S rRNA gene amplicon was amplified using primers 341F-ill (5ʹ-CCTACGGGNGGCWGCAG-3ʹ) and 805R-ill (5ʹ-GACTACHVGGGTATCTAATCC-3ʹ; Klindworth et al., 2013), and the ITS2 region was amplified using primers ITS3 (5ʹ-GCATCGATGAAGAACGCAGC-3ʹ) and ITS4 (5ʹ-TCCTCCGCTTATTGATATGC-3ʹ; Op De Beeck et al., 2014). Equimolar pools of samples were sequenced to generate 250 bp paired-end raw reads in the Novaseq 6000 platform (Illumina). Demultiplexing and trimming of the raw sequences were performed by Novogene.

Trimmed reads for the 16S rRNA and fungal ITS amplicons were processed, aligned, and classified independently using the Divisive Amplicon Denoising Algorithm 2 (DADA2; version 1.26.0) in R studio (version 1.4.1106; Callahan et al., 2016). The forward and reverse read quality profiles were separately examined. To account for the decrease in the quality score of the subsequent nucleotides, the total length of forward and reverse reads was truncated to 220 nucleotides with a maximum error rate of 5 for both forward and reverse reads (truncQ=5) using the ‘filterAndTrim’ function. This function was used to pre-filter sequences in order to remove reads with ambiguous bases for both bacterial and fungal sequences. For the fungal ITS amplicons, the first 10 nucleotides of each read were trimmed to account for the decrease in quality score of the following nucleotides, and a minimum length of 50 nucleotides was enforced to eliminate very short sequences. Reads with ambiguities were removed from both amplicon sets, as were reads that exceeded the probabilistic estimated error of 2 nucleotides for the ITS reads. For both the 16S rRNA and ITS amplicons, amplicon sequence variants were inferred after de-replication of the filtered data and estimation of error rates (Callahan et al., 2016). The inferred forward and reverse sequences were then merged, with paired sequences that did not perfectly match removed to control for residual errors, and a sequence table was constructed. The removeBimeraDenovo () function was used to remove chimeras, and taxonomy was assigned using the SILVA 138.1 ribosomal RNA database for bacteria (Quast et al., 2012) and the UNITE ITS database (version 9.0) for fungi (Nilsson et al., 2019) with a 3% dissimilarity threshold. The raw sequence counts from the taxa tables were collapsed and compositionally normalized such that each sample summed to 1. The relative abundances at the genus rank were statistically analyzed as described below. Alpha-diversity (Shannon, Simpson, observed ASV) analysis was performed using phyloseq (version 1.42.0). For beta-diversity analysis, statistical assessment of dissimilarity matrices (Bray–Curtis) was performed using the ‘adonis2’ function in the R package ‘vegan’ (version 2.6.4; Oksanen et al., 2022), separately for the bacterial and fungal composition. The permutational multivariate analysis of variance (PERMANOVA) was used on the Bray–Curtis distance matrices to assess the dissimilarities between the bacterial and fungal community structures in sows’ feces postpartum and in the gastric and cecal digesta of piglets on the various sampling days during the suckling period. The statistical significance was determined after 999 random permutations. The 2-dimensional non-metric multidimensional scaling (NMDS) ordination plots were generated using the ‘metaMDS’ function and the ggplot2 package was used to visualize the clustering of bacteriomes and mycobiomes in gastric and cecal digesta according to sample type and age as well as between sow feces and digesta samples of piglets.

The datasets generated for this study were deposited into the NCBI Bioproject databank under accession number PRJNA1103974.

### Statistical analyses

The Shapiro-Wilk test with the UNIVARIATE procedure in SAS (version 9.4; SAS Institute, Inc., Cary, NC) was used to test the normal distribution of the residuals of all variables. The residuals were transformed using the Boxcox method and the Transreg procedure in SAS if they were not normally distributed. All data, both from sows, i.e., the gut microbiome (relative abundance of genera), and from piglets, i.e., body weight, the gut microbiome (relative abundance of genera), and fecal score, were subjected to ANOVA using the MIXED procedure in SAS. Across datasets for sow feces and digesta samples from piglets, bacterial >0.2% of all reads and fungal taxa >0.05% of all reads were taken into consideration. The ranked relative abundances were analyzed separately for each genus in SAS. Repeated measures were used to investigate effects on the diversity estimators and relative genera abundances with progressing lactation of the sows (DPP) and increasing age of the piglets (DoL). For data for the diversity estimators and relative genera abundances of the sow, the model included the fixed effects of DPP, replicate batch, litter, and the respective 2- and 3-way interactions. The model for data for the diversity estimators and relative genera abundances of the piglets included the fixed effects of sex, replicate batch, DoL, litter, and the respective 2- and 3-way interactions. For most parameters in piglets, differences between sexes were not detected and were excluded from the final model. For the growth performance parameters of the piglets, the fixed effects were replicate batch, sex, and their interaction, as well as litter size on the weighing day and date of birth were considered covariates. The sow and piglet represented the experimental unit. Degrees of freedom were approximated by the Kenward-Rogers method (ddfm = kr). Data were reported as the least-square means ± standard errors of the mean (SEM). Multiple pairwise comparisons among least-square means were performed using the pdiff statement. A significant difference was defined at *P* ≤ 0.05 and trends at 0.05 < *P* ≤ 0.10. Descriptive statistics using the PROC MEANS procedure in SAS were applied to calculate the feed intake of sows during late gestation and lactation, as well as the creep feed intake of piglets during the suckling period. PROC CORR in SAS was used to calculate Pearson correlation coefficients between cecal bacterial and fungal taxa and postweaning fecal scores on DoL30 and 34. To visualize the obtained correlations, heat maps were generated using the ‘levelplot()’ function in the lattice package in R Studio (version 2023.06.0).

## Results

### Feed intake of sows and piglets and growth performance of piglets

The average feed intake during the late gestation and lactation periods was 3.5 and 6.6 kg/d/sow, respectively (Supplementary Table S3). The creep feed intake was on average 19.6 g/d/piglet during the suckling period (Supplementary Table S4). Except for the higher birth weight of male piglets compared to female piglets (*P* = 0.014; Supplementary Table S5), body weight and average daily weight gain were similar during the suckling and early postweaning period. In total, 23 piglets were removed from the experiment due to illness or died (either crushing or sudden death, mostly in the first week of life).

### Changes in the relative bacterial and fungal abundance and composition in the feces of lactating sows

**Figure 1.**
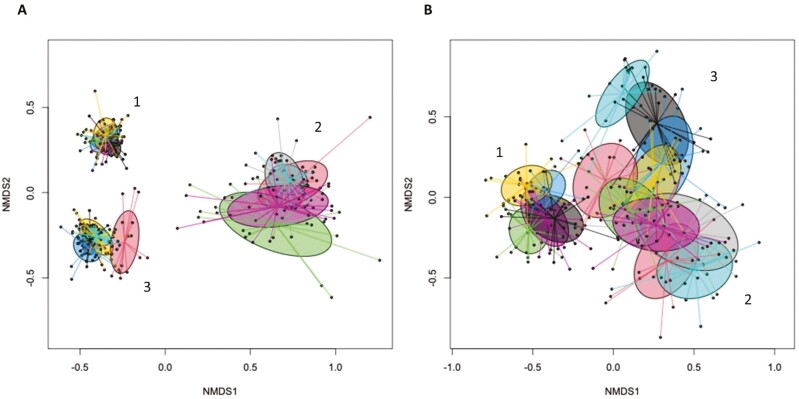
Non-metric multidimensional scaling (NMDS) plot of pairwise Bray–Curtis dissimilarities between communities of bacteriome (A) and mycobiome (B) in sow feces across days postpartum (DPP) and in the gastric and cecal digesta of piglets across days of life (DoL). Ellipses represent the standard deviation. Feces on DPP3 (dark blue), 7 (yellow), 14 (green), 21 (purple), and 28 (dark gray). Gastric digesta on DoL3 (green), 7 (purple), 14 (red), 21 (blue), and 28 (gray). Cecal digesta on DoL3 (red), 7 (blue), 14 (dark gray), 21 (dark blue), and 28 (yellow). Weaning took place on DoL28. Number 1 indicates sow feces, 2 is piglet’s gastric digesta, and 3 is piglet’s cecal digesta.

The PERMANOVA based on Bray–Curtis dissimilarities showed significant separation between animal (i.e., sows and piglets), age (i.e., DoL and DPP), and gut segment (i.e., feces, stomach, and cecum) for the bacterial and fungal communities (*P* < 0.001; Supplementary Table S6). The NMDS (Bray–Curtis dissimilarity) demonstrated separate clustering for the bacterial and fungal communities in sow feces, gastric, and cecal digesta. Time-related clustering of the microbial communities was visible, especially for the communities in gastric and cecal digesta of the piglets but also for the fungal communities in sow feces with increasing DPP (Figure 1A and B).

**Figure 2.**
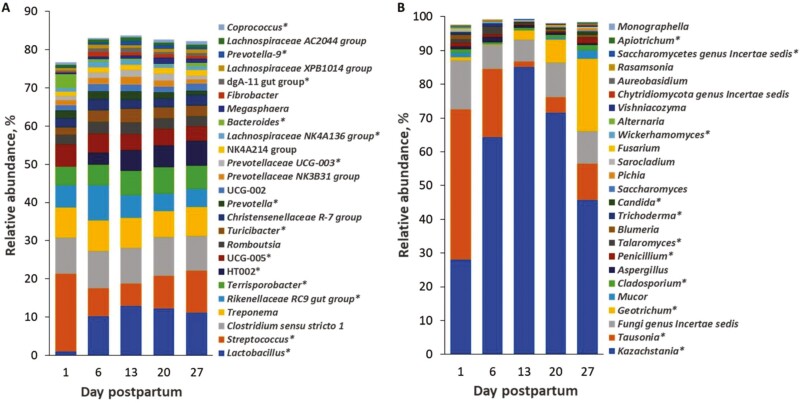
Differences in the relative abundance (%) of the most abundant bacterial (A) and fungal (B) genera in sow feces across days postpartum (*n* = 20/d postpartum). Weaning took place on day 28 postpartum. Effect (*P* < 0.05) of day postpartum is indicated by ‘*’.

Day postpartum did not influence the bacterial species richness (observed ASV) in the feces of sows (Table 1), whereas the bacterial alpha-diversity (Shannon and Simpson) increased from DPP1 to 6 and decreased thereafter until DPP27 (*P* < 0.05). The DPP affected the fungal species richness (observed ASV) and alpha-diversity (Shannon and Simpson; *P *< 0.05; Table 1) but in an opposite manner compared to the bacterial community. The fungal species richness and diversity decreased from DPP1 to 13 but increased thereafter until DPP27 (*P* < 0.05). These observations were supported by the results for the relatively most abundant genera (Figure 2A and B). As an example, the relatively most abundant genus *Lactobacillus* increased by 10.3-fold from DPP1 to 6 (*P* < 0.001), whereas the relative abundance of *Streptococcus* decreased by 3.5-fold from DPP1 to 13 and increased thereafter until DPP27 (*P* < 0.001). The relative abundance of *Rikenellaceae* RC9 gut group increased by 1.6-fold from DPP1 to 6 but decreased afterward on DPP13 by 1.5-fold (*P* < 0.001), whereas the relative abundance of *Terrisporobacter* increased from DPP1 to 20 by up to 1.4-fold (*P* = 0.034). As examples for fungal taxa, the relative abundance of *Kazachstania* as the dominant fungal genus in sow feces increased by 3.0-fold from DPP1 to 13 but decreased thereafter until DPP27 by up to 1.9-fold (*P* < 0.001). The relative abundance of *Tausonia*, which was the dominant genus on DPP1, declined by 28.9-fold from DPP1 to 13 but remained stable thereafter until DPP27 (*P* < 0.001). The relative abundance of *Geotrichum* was low on DPP1 and 6, whereas it increased to DPP27 by up to 20.4-fold, becoming the second dominant fungal genus.

**Table 1.** Development in species richness (Observed ASV) and alpha diversity (Shannon and Simpson) indices for the bacterial and fungal community in sow feces after farrowing (*n* = 20/d postpartum)

Day postpartum	1	6	13	20	27	SEM	*P*-value
Bacterial community							
Observed ASV	1195	1234	1251	1233	1219	37.2	0.909
Shannon	4.96^bc^	5.24^a^	5.14^ab^	5.05^abc^	4.92^c^	0.079	0.020
Simpson	0.957^b^	0.976^a^	0.973^a^	0.969^ab^	0.961^b^	0.004	0.007
Fungal community							
Observed ASV	178^a^	99^b^	95^b^	211^a^	174^a^	23.3	<0.001
Shannon	1.65^a^	1.06^bc^	0.83^c^	1.38^ab^	1.67^a^	0.195	0.007
Simpson	0.546^a^	0.393^ab^	0.265^b^	0.401^ab^	0.549^a^	0.059	0.005

Values are presented as least squares means ± standard error of the mean (SEM). Weaning took place on day 28 postpartum. ASV, amplicon sequence variants.

^a,b,c,d^Means without a common superscript in the same row differ (*P* < 0.05).

### Age-related changes in bacterial and fungal communities in gastric and cecal digesta of piglets

**Figure 3.**
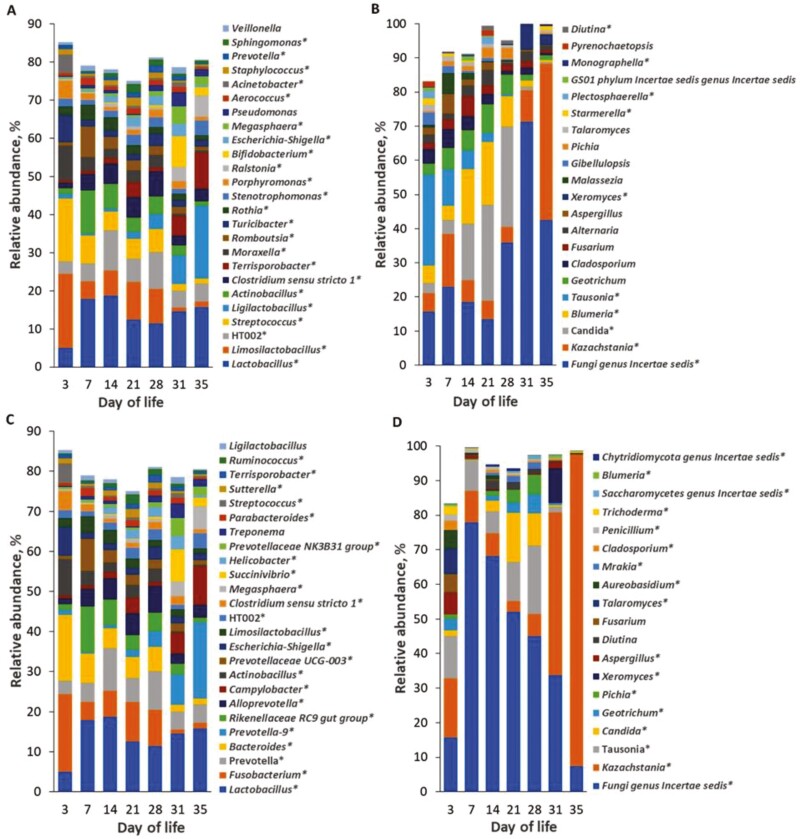
Differences in the relative abundance (%) of the most abundant bacterial genera in gastric (A) and cecal (C) digesta as well as the most abundant fungal genera in gastric (B) and cecal (D) digesta of suckling and newly weaned piglets across days of life (*n* = 20/d of life). Weaning took place on day 28 of life. Effect (*P* < 0.05) of the day of life is indicated by ‘*’.

Day of life did not affect bacterial and fungal species richness (observed ASV) but influenced bacterial (Simpson) and fungal (Shannon, Simpson) diversity (*P* < 0.05) in gastric digesta (Table 2). Accordingly, the bacterial diversity decreased from DoL14 to 31, whereas fungal diversity decreased from DoL28 to 35 in gastric digesta (*P* < 0.05). In cecal digesta, DoL affected bacterial and fungal species richness (observed ASV) and diversity (Shannon, Simpson; *P* < 0.05; Table 2), whereby the effect of the DoL were different for bacteria and fungi. In terms of relative abundances in gastric digesta (Figure 3A), the relative abundances of the predominant *Lactobacillaceae* genera, including *Lactobacillus*, *Limosilactobacillus*, HT002, and *Ligilactobacillus*, together with *Streptococcus* altered from DoL3 to 7 and/or from DoL28 to 31 and 35 (*P* < 0.05). Alterations in relative abundances of fungal genera in gastric digesta were similarly visible (Figure 3B). For instance, gastric relative abundances of *Candida* and *Blumeria* largely increased from DoL7 to 14, whereas those of an unclassified genus and *Kazachstania* greatly increased from DoL28 to 31 (*P* < 0.05).

**Table 2.** Age-related development of species richness (Observed ASV) and alpha diversity (Shannon and Simpson) for the bacterial and fungal communities in the gastric and cecal digesta of suckling and newly weaned piglets (*n* = 20/d of life)

Day of life	3	7	14	21	28	31	35	SEM	*P*-value
Bacterial community in gastric digesta									
Observed ASV	468	434	491	483	897	562	581	132.4	0.180
Shannon	3.54	3.49	3.61	3.38	3.57	3.12	3.08	0.150	0.070
Simpson	0.913^a^	0.907^a^	0.923^a^	0.893^ab^	0.887^ab^	0.860^b^	0.855^b^	0.013	0.002
Fungal community in gastric digesta									
Observed ASV	68	71	58	71	71	68	54	6.2	0.309
Shannon	2.23^ab^	2.47^a^	2.02^b^	2.25^ab^	2.18^ab^	1.88^bc^	1.53^c^	0.135	<0.001
Simpson	0.772^ab^	0.825^a^	0.740^ab^	0.763^ab^	0.758^ab^	0.718^bc^	0.622^c^	0.038	0.020
Bacterial community in cecal digesta									
Observed ASV	749^c^	836^ab^	821^abc^	800^abc^	875^a^	867^ab^	786^bc^	27.7	0.038
Shannon	4.21^b^	4.88^a^	4.87^a^	4.81^a^	4.93^a^	4.72^a^	4.71^a^	0.095	<0.001
Simpson	0.939^b^	0.971^a^	0.974^a^	0.970^a^	0.974^a^	0.962^a^	0.964^a^	0.005	<0.001
Fungal community in cecal digesta									
Observed ASV	240^a^	136^cd^	213^ab^	173^bc^	183^abc^	161^bc^	107^d^	19.8	<0.001
Shannon	3.08^a^	2.74^a^	3.02^a^	2.51^a^	2.54^a^	1.77^b^	0.66^c^	0.201	<0.001
Simpson	0.816^a^	0.866^a^	0.865^a^	0.788^a^	0.790^a^	0.530^b^	0.207^c^	0.048	<0.001

Values are presented as least squares means ± standard error of the mean (SEM). Weaning took place on day 28 of life. ASV, amplicon sequence variants.

^a,b,c,d^Means without a common superscript in the same row differ (*P* < 0.05).

In cecal digesta, the relative abundance of *Fusobacterium* and *Bacteroides* were the predominant bacterial genera on DoL3, whereas the relative abundance of *Lactobacillus* became the dominant genus from DoL7 to 31 (Figure 3C). Postweaning, the relative abundance of *Prevotella*-9 became dominant, whereas the relative abundances of *Fusobacterium* and *Bacteroides* largely dropped compared to the time points during the suckling phase (*P* < 0.05). The relative abundance of *Tausonia* decreased postweaning compared to the suckling phase, whereas the relative abundance of *Kazachstania* largely increased from DoL28 to 35 (*P* < 0.001; Figure 3D).

### Postweaning fecal scores

**Figure 4.**
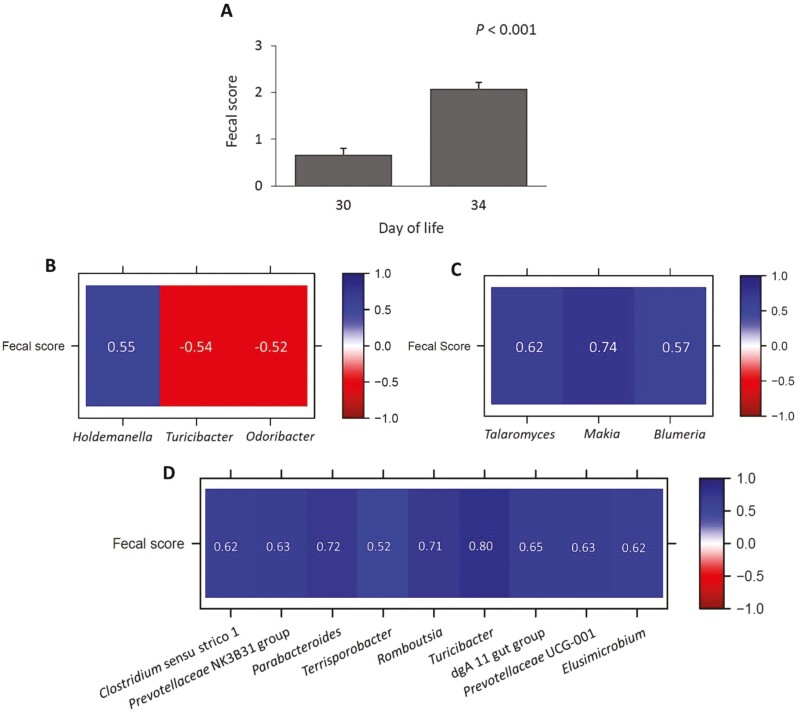
Differences in fecal scores of weaned piglets between days 30 and 34 of life (A; *n* = 110 piglets). Pearson’s correlation heat map shows significant associations (*P* < 0.05) between fecal scores of the weaned piglets from which gut digesta was collected and cecal bacterial genera (B) on day 30 of life (*n* = 20 piglets), as well as between fecal scores and cecal fungal (C) and bacterial (D) genera on day 34 of life (*n* = 20 piglets). The fecal scoring system consisted of 0 (hard and dry balls), 1 (clearly defined shape with cracks), 2 (pasty; moist feces with no cracks), 3 (very wet but not liquid feces), 4 (liquid feces with minimal consistency), and 5 (entirely liquid feces). Piglets were weaned on day 28 of life. Fecal score values are least squares mean and standard error of the mean.

Feces for scoring were obtained from 110 piglets each on DoL30 and 34. The fecal score was 0.67 on DoL30 and increased by 3.1-fold on DoL34 (*P* < 0.001; Figure 4A). Fecal scores of the piglets (n = 20) from which gut samples were obtained were correlated with the cecal bacterial abundances. Fecal scores correlated positively (*P* < 0.05) with the relative abundance of *Holdemanella,* (*r *= 0.55) and negatively with the relative abundance of *Turicibacter (r *= −0.54) and *Odoribacter (r = *−0.52) but not with fungal genera on DoL30 (Figure 4B). On DoL34, the fecal score positively correlated with the relative abundance of 3 fungal genera (*Talaromyces*, *Mrakia*, and *Blumeria* (*r* > 0.5; *P* < 0.05; Figure 4C), as well as with that of 9 bacterial genera (*Clostridium* sensu stricto 1, *Prevotellaceae* NK3B31 group, *Parabacteroides*, *Terrisporobacter*, *Romboutsia*, *Turicibacter*, dgA-11 gut group, *Prevotellaceae* UCG-001, and *Elusimicrobium* (*r* > 0.5; *P* < 0.05; Figure 4D).

## Discussion

The present study provides novel information about the alterations of the bacterial and fungal communities in sow feces during the lactation period as well as about the fungal diversity that establishes in the piglet’s gastrointestinal tract after birth. There was a large overlap in bacterial and fungal taxa between sow feces and gastric and cecal digesta of piglets during the suckling period, especially in the early days postpartum, which supports that the microbial community in sow feces is important for the neonatal gut colonization of piglets. Notably, the large variety of fungal taxa present and their relative abundance in the gastric and cecal digesta of the piglets on DoL3 resembled to a certain degree the fungal abundances in sow feces on DPP1. The uptake of feces by the piglets is similar to the concept of ‘fecal transplants’ (McCormack et al., 2018). Therefore, our results may be useful for future formulation of transition and lactation diets for sows to modulate their fecal microbiome in order to target specific developmental stages in the gut microbiome of suckling piglets. Nevertheless, the differences in relative abundances indicated that the establishment of the microbes in the gut segments depended on the ‘local’ conditions (e.g., lower stomach pH in the stomach and nutrient availability) and other sources of microbes (e.g., sow colostrum/transient milk, skin, and environment). This assumption was supported by the beta-diversity, demonstrating different clustering bacterial and fungal communities for sow feces, and gastric and cecal digesta of piglets. With regard to environmental microbes, the milk replacer was based on bovine whey, which probably contained microbes from cheese-making that could have contributed to gut colonization.

Different types of fungi were present in sow feces and piglets’ digesta including common gut inhabitants (e.g., *Kazachstania*; Harlow et al., 2024), plant-related fungi (Pietrusińska and Tratwal, 2020), yeasts (e.g., *Candida*; Pérez, 2021) and mold fungi (Liew and Mohd-Redzwan, 2018). Metabolically, these fungi probably filled different niches, breaking down residual dietary carbohydrates (Luo et al., 2021) as well as being involved in epithelial glucose turnover (Hu et al., 2023). Some plant-related fungi—some of them plant pathogens (e.g., *Blumeria*; Mapuranga et al., 2022)—may have been transient, colonizing the plant feed particles. Their role in the gut microbe-microbe/host interactions needs further research. Furthermore, the abundance of *Cladosporium, Alternaria, Fusarium,* and *Aspergillus* in sow feces and piglet’s digesta showed that the animals may have been exposed to mold and ergot fungi from the environment and feed components (i.e., milk replacer and prestarter diet, lactation diet and hay) and hence potentially to mycotoxins and ergot alkaloids which may compromise their health (Coufal-Majewski et al., 2016; Deligeorgakis et al., 2023).

Results for the beta-diversity and ANOVA demonstrated that the bacterial and fungal communities in sow feces were not stable throughout lactation but continuously changed in relative abundance and diversity. This was probably a result of stress and dietary changes (i.e., discontinuation of feeding linseeds) around farrowing as well as the gradual increase in feed intake level during lactation (Jašarević et al., 2017; Lu et al., 2022). More specifically, the bacterial community changed more between DPP1 and 6, whereas the 4 dominant fungal taxa showed large changes in their abundances throughout the lactation phase. The fungal community was more susceptible to changes in fermentable substrate quality and quantity that reached the distal parts of the hindgut and/or substrate-related microbial interactions with progressing lactation than the bacterial community. Sows had access to hay as environmental enrichment; as a slowly fermentable fiber, it likely acted as a substrate for fibrolytic bacterial (e.g., *Treponema* and *Fibrobacter*; Xie et al., 2018) and fungal taxa (e.g., *Mucor;*Karimi and Zamani, 2013) in feces. Unfortunately, we do not have exact data for the hay intake of sows. Of note, the species richness and diversity of the fungi were greater in the cecal digesta of piglets than in cow feces in the first 2 wk of life, which may be linked to the immature immune system and microbe-to-microbe interactions.

The piglets showed a similar increase in *Lactobacillaceae* in gastric and cecal digesta to sow feces from DoL3 to 7, which may be advantageous due to their multiple beneficial effects on the control of gut homeostasis and immune development (Valeriano et al., 2017; Zhang et al., 2022). From the relative abundance patterns, it can be assumed that sow feces were not the only source of *Lactobacillaceae* for the colonization of the piglet’s stomach and distal gut. However, their predominance throughout the suckling phase was likely supported by the milk glycans. Of note, *Lactobacillus* was similarly relatively abundant in gastric and cecal digesta, whereas other milk glycan fermenters, such as *Limosilactobacillus*, HT002, and *Ligilactobacillus*, *Actinobacillus, Fusobacterium* and *Bacteroides* (García-Alija et al., 2022), were differently abundant in gastric and cecal digesta. Different substrate availabilities and subsequent cross-feeding relationships were probably behind these observations, also explaining the higher bacterial diversity in cecal compared to gastric digesta. Moreover, the bacterial and fungal composition probably reflected the slowly increasing amounts of creep feed as well as the change in the type of creep feed from the milk replacer to the prestarter from DoL21 to 28. Even in very small amounts plant glycans, such as starch, probably supported the growth of starch-degrading taxa such as *Turicibacter*, *Terrisporobacter*, *Porphyromonas,* and *Prevotella* in digesta (Sun et al., 2015; Umu et al., 2015; Trachsel et al., 2019). As an example, the starch component may have proportionally lowered the relative abundance of milk glycan-fermenting bacteria in gastric digesta on DoL21. Other bacteria, such as *Clostridium* sensu stricto, may have thrived on milk peptides, either from sow milk or milk replacer.

*Kazachstania* is a commensal yeast in the porcine gut (Summers et al., 2021), which per se may explain its high presence in sow feces and gastric and cecal digesta of piglets. They feed on dietary or host-derived sugars, such as glucose and galactose (Kondybayev et al., 2023). Previously, *Kazachstania slooffiae* has been linked to the provision of amino acids and energy to other bacteria, such as *Lactobacillus* and *Prevotella*, as well as the host piglet (Summers et al., 2021; Hu et al., 2023). The relative abundance of the yeast *Candida* increased more in gastric than in cecal digesta of piglets during the suckling phase, which may be linked to the glycan intake from milk and creep feed. *Geotrichum*, which was another relatively abundant fungus in sow feces but low abundant in gastric and cecal digesta of the piglets, may exert some positive effects on the host. In this respect, *Geotrichum* has been used as a probiotic in ruminant nutrition to promote productivity and bacterial diversity in dairy cattle (Zaman et al., 2022), whereas very little is known for pigs. According to its abundance pattern in feces throughout lactation, the abundance of *Geotrichum* seemed to be related to the feed intake level of the sows.

The diversity and relative abundance patterns of the bacterial and fungal communities allowed distinguishing the time point of weaning. It can be assumed that the weaning-related removal of sow milk and low feed intake as well as changes in the bacterial crosstalk may be behind the decreasing fungal diversity in cecal digesta from DoL28 (preweaning) to DoL35 (postweaning). It is difficult to relate the changes in the fungal community postweaning to the action of certain bacteria as our understanding is only advancing. Weaning changed the relative bacterial and fungal abundances in the sense of milk glycan fermenters being replaced by plant glycan utilizers. For instance, the removal of the milk glycans reduced the abundance of *Limosilactobacillus* and HT002 in gastric digesta. By contrast, other *Lactobacillaceae*, such as *Lactobacillus* and *Ligilactobacillus,* in gastric and cecal digesta remained stable or increased in their abundance postweaning, indicating that they were capable of utilizing the glycans in the pre-starter diet (i.e., starch and lactose) and/or those derived from the host. Of note, *Bifidobacterium*, another genus known for its milk glycan-fermenting capabilities (Jang and Kim, 2022), only raised in its abundance in gastric digesta on DoL31. This taxon possibly benefited from the opening niches of *Lactobacillaceae* but seemed to be outcompeted again on DoL35, e.g., by HT002 and *Ligilactobacillus*. In cecal digesta, typical plant glycan-utilizing taxa, like *Prevotellaceae* genera and *Succinivibro* (Tan and Nie, 2020), became dominant on DoL31 and 35. Regarding the fungi, *Kazachstania* potentially benefited from increased glucose release from starch fermentation or mucosal glycolysis in both gastric and cecal digesta. From the abundance of pathobionts, the increase in *Campylobacter* in cecal digesta postweaning may be worth mentioning. Although it mostly does not cause enteritis in pigs, *Campylobacter* is an important zoonotic agent that is shed in feces, with the potential to be transmitted to humans (Ishengoma et al., 2023). By contrast, the gut conditions postweaning seemed to lower the relative abundance of mold and ergot fungi in gastric and cecal digesta. The piglets from which the gut samples were collected were not diarrhetic, as indicated by the fecal scores. This may be the reason for the few relationships between cecal microbial genera and fecal consistency on DoL30. Piglets had firm feces on DoL30. Accordingly, the fecal score only moderately correlated with 3 starch-fermenting and short-chain fatty acid-producing bacteria, namely *Holdemanella*, *Turicibacter,* and *Odoribacter,* but not with *Campylobacter*. On DoL34, only the positive association between fecal score and *Clostridium* sensu stricto 1 may relate moister feces to bacterial toxins. The other positive correlations with bacterial taxa (e.g., *Prevotellaceae* NK3B31 group, *Prevotellaceae* UCG-001, and *Terrisporobacter*) may have been indicative of increased complex carbohydrate fermentation and short-chain fatty acid production in the large intestine, which are osmotically active and increase the fecal water content (Amat et al., 2020; Niu et al., 2023). The 3 identified positive correlations of the fecal score with fungi were only with low abundant genera. Nevertheless, as these taxa were mold and plant pathogenic fungi, an activation of secretory functions in the large intestine may be thinkable.

In conclusion, this study demonstrated that progressing lactation affected bacterial and fungal community structure and bacterial richness in sows. Our results also support that sow feces were a contributing source of microbes for the gut colonization of piglets, as indicated by the shared bacterial and fungal taxa in sow feces and in the gastric and cecal digesta of piglets. We could identify microbial taxa in cecal digesta that are associated with the fecal scores on DoL30 and DoL34. However, fecal scores indicated that the feces were firm to moist but not entirely liquid feces. Therefore, the relationships miss information for a low gut homeostatic state postweaning.

## Supplementary Material

skae321_suppl_Supplementary_Tables_S1-S6

## Abbreviations

ASV: amplicon sequence variant
DADA2: Divisive Amplicon Denoising Algorithm 2
DoL: day of life
DPP: day postpartum
NMDS: non-metric multidimensional scaling
pdiff: probability of difference
PERMANOVA: permutational multivariate analysis of variance
w/v: weight/volume
w/w: weight/weight
