# Supplementary material for: Lactation-related dynamics of bacterial and fungal microbiomes in feces of sows and gut colonization in suckling and newly weaned piglets
Source: J Anim Sci. 2024 Oct 26;102:skae321. doi: 10.1093/jas/skae321 (PMC11604110; doi:10.1093/jas/skae321)
Supplement: skae321_suppl_Supplementary_Tables_S1-S6 [file skae321_suppl_supplementary_tables_s1-s6.docx]

**Lactation-related dynamics of bacterial and fungal microbiomes in feces of sows and gut colonization in suckling and newly weaned piglets**

Fitra Yosi,^*,#,†^ Frederike Lerch,^*,#^ Julia C. Vötterl,^*,#^ Simone Koger,^#,§^ Doris Verhovsek,^¶^ and Barbara U. Metzler-Zebeli^*,#,1^

^*^Centre for Veterinary Systems Transformation and Sustainability, Clinical Department for Farm Animals and Food System Science, University of Veterinary Medicine Vienna, Vienna 1210, Austria.

^#^Christian-Doppler Laboratory for Innovative Gut Health Concepts of Livestock, Institute of Animal Nutrition and Functional Plant Compounds, Centre for Animal Nutrition and Welfare, University of Veterinary Medicine Vienna, Vienna 1210, Austria.

^†^Department of Animal Science, Faculty of Agriculture, University of Sriwijaya, Palembang 30662, Indonesia.

^§^ Centre for Animal Nutrition and Welfare, Clinical Department for Farm Animals and Food System Science, University of Veterinary Medicine Vienna, Vienna 1210, Austria.

^¶^ Clinical Centre for Population Medicine in Fish, Pig and Poultry, Clinical Department for Farm Animals and Food System Science, University of Veterinary Medicine Vienna, Vienna 1210, Austria.

^1^Corresponding author: [barbara.metzler@vetmeduni.ac.at](mailto:barbara.metzler@vetmeduni.ac.at)

**Supplementary Materials:**

Table S1. Ingredient composition of gestation, lactation and prestarter diets

| Ingredients, % | Gestation diet^1^ | Lactation diet^2^ | Prestarter^3^ |
| --- | --- | --- | --- |
| Barley meal | 50.0 | 20.0 | 29.7 |
| Wheat bran | 15.8 | 6.4 | – |
| Corn meal | 10.0 | 29.0 | 10.8 |
| Wheat meal | 8.1 | 10.0 | 10.0 |
| Soybean meal | 4.2 | 9.3 | – |
| Fullfat soy | – | – | 14.1 |
| Wheat pressure cooked | – | – | 9.9 |
| Sweet whey powder | – | – | 3.0 |
| Potato protein | – | – | 5.0 |
| Rapeseed meal | – | 5.0 | – |
| Dried pulp | 4.0 | 1.5 | – |
| Commercial breeding premix | 2.8 | 3.5 | – |
| Lignocellulose | – | 0.5 | 0.9 |
| Palm kernel | – | – | 2.0 |
| Dried vinasse^4^ | 1.5 | – | 1.9 |
| Dextrose | – | – | 5.0 |
| Lactose | – | – | 3.0 |
| Apple pomace | 1.5 | 1.0 | – |
| Bakery products | 1.2 | 12.8 | – |
| Hay cobs | 0.5 | – | – |
| Oil | 0.3 | 0.5 | – |
| Rapeseed oil | – | – | 0.5 |
| Lysine HCl | – | 0.2 | 0.7 |
| Threonine | – | 0.06 | 0.3 |
| Methionine | – | 0.02 | 0.3 |
| Tryptophane | – | – | 0.1 |
| Limestone (calcium carbonate) | – | 0.2 | 0.6 |
| Sodium chloride | 0.03 | – | 0.5 |
| Mono calcium phosphate | – | – | 1.4 |
| Magnesium phosphate | – | – | 0.2 |
| Vitamin E | 0.02 | 0.02 | – |
| Vitamin/trace element premix | – | – | 0.4 |

^1^Vitamin and mineral composition per kg feed: 9,600 IE of vitamin A, 1,600 IE of vitamin D3, 156 mg of vitamin E, 82 mg of Fe as iron(II) sulfat monohydrate, 12 mg of Cu as copper(II) sulfat pentahydrate, 90 mg of Zn as zinc oxide, 2.6 mg of Mn as manganese(II) oxide. Technological additives: 700 FTU of 6-phytase, 60 mg of butylated hydroxytoluene.

^2^Vitamin and mineral composition per kg feed: 12,000 IE of vitamin A, 2,000 IE of vitamin D3, 170 mg of vitamin E, 100 mg of Fe as iron(II) sulfat monohydrate, 15.1 mg of Cu as copper(II) sulfat pentahydrate, 110 mg of Zn as zinc oxide, 0.7 mg of Mn as manganese(II) oxide. Technological additives: 880 FTU of 6-phytase, 70 mg of butylated hydroxytoluene.

^3^Vitamin and mineral composition per kg feed: 16,000 IE of vitamin A, 2,000 IE of vitamin D3, 150 mg of vitamin E, 4.0 mg of vitamin K3, 2.8 mg of vitamin B1, 8.2 mg of vitamin B2, 5.0 mg of vitamin B6, 50 mg of vitamin B12, 60 mg of nicotinic acid, 20 mg of panthothenic acid, 500 mg of cholin chloride, 1,050 mcg of folic acid, 150 mcg of biotin, 124 mg of Fe as iron(II) sulfat monohydrate, 80 mg of Mn as manganese(II) oxide, 3.1 mg of I as calcium iodate, 121 mg of Zn as zinc oxide, 0.45 mg of Se as sodium selenite, 124 mg of Cu as copper(II) sulfat pentahydrate. Technological additives: 250 FTU of phytase (4a16), 100 mg of butylated hydroxytoluene.

^4^CITROFEED, dried residues from citric acid production.

**Table S2**. Analyzed nutrient composition of gestation and lactation diets for sows as well as the milk replacer and prestarter diet for piglets

| Chemical composition, % dry matter | Gestation diet | Lactation diet | Milk replacer^1,2^ | Prestarter diet^2^ |
| --- | --- | --- | --- | --- |
| Dry matter, % | 90.4 | 89.3 | 91.6 | 89.8 |
| Crude protein | 14.7 | 17.8 | 23.3 | 18.2 |
| Crude fiber | 6.5 | 4.6 | 1.9 | 4.9 |
| Ether extract | 5.1 | 4.9 | 10.3 | 7.3 |
| Crude ash | 5.7 | 6.6 | 6.8 | 5.1 |
| Nitrogen-free extract | 68.2 | 66.2 | 57.9 | 65.0 |
| Starch | 44.3 | 46.7 | 25.4 | 42.5 |
| Metabolizable energy, MJ/kg | 14.2 | 14.9 | 16.9 | 15.4 |
| Macro minerals, % |  |  |  |  |
| Calcium | 0.89 | 1.20 | 0.80 | 0.73 |
| Phosphorus | 0.63 | 0.67 | 0.80 | 0.72 |
| Magnesium | 0.23 | 0.26 | 0.20 | 0.21 |
| Potassium | 0.78 | 0.78 | 1.09 | 0.67 |
| Sodium | 0.28 | 0.36 | 0.50 | 0.24 |
| Trace minerals, ppm |  |  |  |  |
| Iron | 264.0 | 324.7 | 264.2 | 333.2 |
| Manganese | 95.2 | 96.9 | 87.3 | 118.1 |
| Zinc | 142.2 | 166.9 | 128.8 | 181.7 |
| Copper | 18.3 | 24.1 | 146.3 | 152.1 |

^1^NuriStart Sweet, BIOMIN Holding GmbH, Part of dsm-firmenich, Getzersdorf, Austria. Ingredients: Wheat flour, whey protein concentrate, whey powder, extruded soybeans, puffed corn, rolled oats, soy protein concentrate, sugar, dextrose, palm oil, monocalcium phosphate,

coconut oil, sodium chloride, calcium carbonate, and salmon oil. Technological additives per kg: 400 mg sepiolite (E562), 3,000 mg citric acid (E330), 0.07mg propyl gallate (E310), 1 mg butyrylhydroxy toluol (E321). Zootechnical additives per kg: 200 FXU endo-1,4-beta-xylanase EC 3.2.1.8; 1,000 FYT 6-phytase EC 3.1.3.26. Nutritional additives per kg: 16,000 IE of vitamin A, 2,000 IE of vitamin D3, 150 mg vitamin E, 200 mg vitamin C, 195 mg Fe as iron (II) sulfate monohydrate, 2.0 mg I as calcium iodate anhydrous, 0.40 mg Se as sodium selenite, 60 mg Mn as manganese (II) oxide, 140 mg Cu as copper (II) sulfate pentahydrate, 100 mg Zn as zinc oxide.

^2^Piglets were fed with 100% milk replacer from day of life (DoL) 3 to 23; combinations milk replacer and prestarter diet with a ratio of 70:30 (w/w) on DoL24, 50:50 (w/w) on DoL25, and 30:70 (w/w) on DoL26, respectively; and 100% prestarter diet on DoL27 and 28.

**Table S3.** Descriptive statistics for average daily feed intake of sows (n=20) during the gestation and lactation period

| Average daily feed intake, kg/DM^1^ | Mean | SD |
| --- | --- | --- |
| Pre-farrowing (gestation period) |  |  |
| 26 to 5 days before farrowing | 3.5 | 1.30 |
| 5 days before farrowing | 2.5 | 0.43 |
| Post-farrowing (lactation period)^2^ |  |  |
| Days 1 to 7 | 3.8 | 0.95 |
| Days 8 to 14 | 6.3 | 1.68 |
| Days 15 to 21 | 8.2 | 1.22 |
| Days 22 to 28 | 8.3 | 1.93 |

DM, dry matter; SD, standard deviation.

^1^Sows were fed with gestation diet from 26 to 5 days prior to farrowing, and the lactation diet from 5 days before farrowing until 28 days postfarrowing.

^2^Mean values for the feed intake postfarrowing are provided. The feed intake that was offered to the sows was gradually increased in the first three weeks of lactation.

**Table S4.** Descriptive statistics for average daily creep feed intake of piglets during the suckling period

| Average daily feed intake, g/DM^1,2^ | Mean | SE |
| --- | --- | --- |
| DoL3 to 9 | 19.7 | 1.14 |
| DoL10 to 16 | 16.5 | 0.77 |
| DoL17 to 23 | 20.9 | 0.88 |
| DoL24 to 26 | 23.5 | 1.79 |
| DoL27 to 28 | 17.4 | 1.92 |

DM, dry matter; DoL, day of life; SE, standard error.

^1^Creep feed intake was estimated on litter basis.

^2^Piglets were fed with 100% milk replacer from DoL3 to 23; combinations of milk replacer and prestarter diet with a ratio of 70:30 (w/w) on DoL24, 50:50 (w/w) on DoL25, and 30:70 (w/w) on DoL26, respectively; and 100% prestarter diet on DoL27 and 28.**Table S5.** Body weight and average daily gain of suckling and newly weaned piglets

| Item | Female | Male | SEM | *P*-value |
| --- | --- | --- | --- | --- |
| Body weight, kg | | | | |
| Birth | 1.4^b^ | 1.5^a^ | 0.032 | 0.014 |
| DoL4 | 1.7 | 1.7 | 0.016 | 0.678 |
| DoL6 | 2.1 | 2.1 | 0.015 | 0.140 |
| DoL13 | 3.7 | 3.7 | 0.048 | 0.861 |
| DoL20 | 5.5 | 5.4 | 0.055 | 0.207 |
| DoL27 | 7.2 | 7.3 | 0.065 | 0.619 |
| DoL30 | 7.1 | 7.2 | 0.041 | 0.289 |
| DoL34 | 7.4 | 7.3 | 0.038 | 0.434 |
| Average daily gain, kg | | | | |
| DoL1 to 4 | 0.13 | 0.13 | 0.006 | 0.902 |
| DoL4 to 6 | 0.19 | 0.20 | 0.007 | 0.186 |
| DoL1 to 6 | 0.16 | 0.16 | 0.006 | 0.210 |
| DoL6 to 13 | 0.23 | 0.23 | 0.007 | 0.823 |
| DoL13 to 20 | 0.25 | 0.24 | 0.008 | 0.255 |
| DoL20 to 27 | 0.27 | 0.27 | 0.010 | 0.588 |
| DoL27 to 30 | 0 | 0 | 0.026 | 0.782 |
| DoL30 to 34 | 0.05 | 0.03 | 0.031 | 0.630 |
| Average daily gain: birth to day 34 of life, kg | | | | |
| DoL1 to 27 | 0.24 | 0.24 | 0.008 | 0.795 |
| DoL28 to 34 | 0.02 | 0.01 | 0.021 | 0.647 |
| DoL1 to 34 | 0.18 | 0.18 | 0.007 | 0.986 |

Values are presented as least squares means ± standard error of the mean (SEM) of all piglets on trial. DoL, day of life. Piglets were weaned on DoL28.

^a,b^Means without a common superscript in the same row differ (*P* < 0.05).

**Table S6**. Permutational multivariate analysis of variance (PERMANOVA) results for dissimilarities in the bacterial and fungal communities in sow feces and gastric and cecal digesta of piglets during the suckling phase

| Source of variation | df | Sum of squares | R^2^ | F | *P*-value |
| --- | --- | --- | --- | --- | --- |
| Bacterial community | | | | | |
| Day × animal × gut segment^1^ | 14 | 36.93 | 0.651 | 36.013 | 0.001 |
| Residual | 270 | 19.78 | 0.349 |  |  |
| Total | 284 | 56.71 | 1.000 |  |  |
| Fungal community | | | | | |
| Day × animal × gut segment^1^ | 14 | 30.38 | 0.444 | 13.245 | 0.001 |
| Residual | 232 | 38.01 | 0.556 |  |  |
| Total | 246 | 68.39 | 1.000 |  |  |

The analysis based on pairwise distance of a multivariate data set and values were obtained using type III sums of squares with 999 permutations of residuals, considering significant difference at *P* < 0.05; df, degrees of freedom; F, F-value by permutation.

^1^Day, days of life for piglets and days postpartum for sows; animal, piglet and sow; gut segment, sow’s feces and gastric and cecal digesta of piglets.
